# Evidence of Rehabilitative Impact of Progressive Resistance Training (PRT) Programs in Parkinson Disease: An Umbrella Review

**DOI:** 10.1155/2020/9748091

**Published:** 2020-05-26

**Authors:** T. Paolucci, S. Sbardella, C. La Russa, F. Agostini, M. Mangone, L. Tramontana, A. Bernetti, M. Paoloni, L. Pezzi, R. G. Bellomo, V. Santilli, R. Saggini

**Affiliations:** ^1^University G. D'Annunzio Chieti, Department of Medical and Oral Sciences and Biotechnologies, Chieti-Pescara, Italy; ^2^Department of Anatomy, Histology, Forensic Medicine and Orthopedics, Board of Physical Medicine and Rehabilitation, “Sapienza” University, Rome, Italy; ^3^S. Filippo Neri Hospital, Physical Medicine and Rehabilitation, “Sapienza” University, Rome, Italy; ^4^Department of Anatomy, Histology, Forensic Medicine and Orthopedics, Board of Physical Medicine and Rehabilitation, “Sapienza” University, Policlinico Umberto I, Rome, Italy; ^5^University of Study of Urbino Carlo Bo, Department of Biomolecular Sciences, Urbino, Italy

## Abstract

Parkinson disease (PD) is a chronic neurodegenerative condition that leads to progressive disability. PD-related reductions in muscle strength have been reported to be associated with lower functional performance and balance confidence with an increased risk of falls. Progressive resistance training (PRT) improves strength, balance, and functional abilities. This umbrella review examines the efficacy of PRT regarding muscular strength in PD patients. The PubMed, PEDro, Scopus, and Cochrane Library databases were searched from January 2009 to August 2019 for systematic reviews and meta-analyses conducted in English. The populations included had diagnoses of PD and consisted of males and females aged >18 years old. Outcomes measured were muscle strength and enhanced physical function. Eight papers (six systematic reviews and meta-analyses and two systematic reviews) were considered relevant for qualitative analysis. In six of the eight studies, the reported severity of PD was mild to moderate. Each study analyzed how PRT elicited positive effects on muscle strength in PD patients, suggesting 10 weeks on average of progressive resistance exercises for the upper and lower limbs two to three times per week. However, none of the studies considered the postworkout follow-up, and there was no detailed evidence about the value of PRT in preventing falls. The possibility of PRT exercises being effective for increasing muscle strength in patients with PD, but without comorbidities or severe disability, is discussed. Overall, this review suggests that PRT should be included in rehabilitation programs for PD patients, in combination with balance training for postural control and other types of exercise, in order to preserve cardiorespiratory fitness and improve endurance in daily life activities.

## 1. Introduction

Parkinson disease (PD) is a progressive neurodegenerative chronic disease that is characterized by tremor, muscle rigidity, and bradykinesia [1]. Patients can present with postural instability, leading to an increased risk of falls, social isolation, and a decline in quality of life [2]. PD is characterized by Lewy bodies containing alpha-synuclein and a reduction in dopamine concentrations in the substantia nigra. Additionally, PD involves dysfunctional cholinergic transmission due to neuronal loss in the nucleus basalis of Meynert and noradrenergic and serotoninergic ascending systems [3]. These impairments result in neurovegetative disorders, mood disturbances, and cognitive dysfunction [4]. PD affects 1-2 per 1000 persons in the population at any time, and its prevalence increases with age, affecting 1% of those aged over 60 years. In 90% of PD cases, the cause is sporadic, whereas the remaining 10% of PD cases involve mutations in one or more genes, which may lead to earlier onset [5]. It is important to emphasize how PD negatively impacts quality of life due to increased motor disability, loss of independence, and social isolation [2]. In the early stages, due to the greater risk of falls, patients limit their ambulation out of the home [6] and their participation in outdoor activities [7].

The value of rehabilitation is thus fundamental, in combination with drug therapy, to preserve functional ability and a minimum level of autonomy through simple and complex Activities of Daily Life (ADLs) [8]. An integrated rehabilitative program with physical activities, therapeutic exercises, and fall prevention strategies could slow the progression of disability [9]. Innovative techniques, such as virtual reality (VR), motor imagery (MI), action observation therapy (AOT), and robot-assisted physiotherapy, have been used recently [10] with good results and good compliance by patients. New technologies could improve motor performance and promote the learning of motor tasks [11]. However, physical activity has a positive effect on both motor and nonmotor symptoms in PD and is beneficial, cost-effective, and low-risk [12], with neuroprotective and neurorestorative effects [13]. For example, Bhalsing et al. recommend physical activity in PD, considering a patient's specific factors (motor symptoms, risk of fall, apathy, fatigue, depression, and cognitive dysfunction) [12], and Fayyaz et al. showed that “physical exercise” can be used as an adjuvant treatment to help a PD patient's limitations [14]. Subsequent therapeutic exercise improves transfers, gait cycle, balance, aerobic endurance, and the early stage of movements [8], especially in the initial phases of PD.

Practitioners prescribe physical therapy coupled with drugs and cognitive treatments [15] because cognitive difficulties often increase disabilities, especially impaired attention [16]. In the physical exercise program designed to prevent falls, resistance training (RT) and endurance training (ET) enhance muscle strength and improve range of motion, muscle power, and balance [17]. An effective physical therapy method is “*movement strategy training*” (MST), which teaches patients to use compensation strategies to improve their motor abilities and cognitive resources in order to initiate and execute functional activities [18].

Similarly, progressive resistance training (PRT) can enhance muscle strength, which is frequently reduced in PD due to bradykinesia and disuse of muscles for immobilization [19]. PRT was developed in the 1940s to rehabilitate veterans of World War II. After that, it became an efficient treatment for rehabilitating young people and athletes, improving pain and muscle strength [20]. Recent data describe PRT as an effective rehabilitation method in PD, improving joint mobility, endurance, and performance in daily living activities [21]. Moreover, PRT increases endurance during gait training [22], prevents falls [8], and might have beneficial effects on nonmotor symptoms, such as cardiovascular autonomic dysfunction [23], which are often disabling in PD patients. Unilateral PRT optimizes improvements in strength in the contralateral limb, “*cross-education phenomenon*” [24]. Further, PRT improves sensorimotor coordination, leading to adjustments in the areas that control voluntary movements [25].

Based on these premises and the increasing number of systematic reviews on the efficacy of PRT in PD, we performed this umbrella review to highlight the specific goals of PRT in rehabilitation in PD and examine the efficacy of PRT regarding muscular strength in PD patients.

## 2. Materials and Methods

The PICO (Population, Intervention, Comparison, and Outcome) method was used to arrange this review. *Population:* eligible trials involved patients with Parkinson disease, regardless of gender or level of disability. *Interventions:* the experimental intervention was PRT exercises, deﬁned as repetitive muscle contractions against increasing load, based on the patient's abilities. *Comparison:* reviews that compared PRT with placebo, no treatment, or another treatment (such as endurance training) were included. *Outcome measures:* the effect of PRT exercises on muscular strength was evaluated.

### 2.1. Inclusion Criteria

We included systematic reviews and meta-analyses of randomized and other controlled studies from the past ten years that compared PRT with placebo or another form of exercise in PD. Selected populations had a diagnosis of PD at any level of severity, comprising males and females aged >18 years old. Outcomes measured were muscle strength and enhancement in physical function.

### 2.2. Exclusion Criteria

We excluded all randomized controlled trials (RCTs) or experimental studies or reviews that were published until August 2019, articles that were not in English, those that did not have the full text available, and articles about other interventions not involving PRT (Table 1).

### 2.3. Data Sources and Search Strategy

Three databases were searched by three independent reviewers in PubMed, PEDro, Scopus, and Cochrane Library with the following filters: “systematic review,” “meta-analysis,” and “practice guidelines.”

The search was based on reviews from 2009 to August 2019 that were written in English and based on a high level of evidence (systematic reviews and meta-analyses). The keywords were “Parkinson disease,” “progressive resistance training,” “resistance training,” and “muscle strength.”

### 2.4. Methodological Quality

Methodological quality/bias risk was recorded using the Joanna Briggs Institute critical appraisal checklist for Systematic Reviews and Research Syntheses [26]. For data extraction, three investigators carried out the research autonomously, subsequently crossed the data to screen titles and abstracts, and independently assessed the risk of bias. Disputes were resolved by consensus (Table 2).

## 3. Results

**Figure 1:**
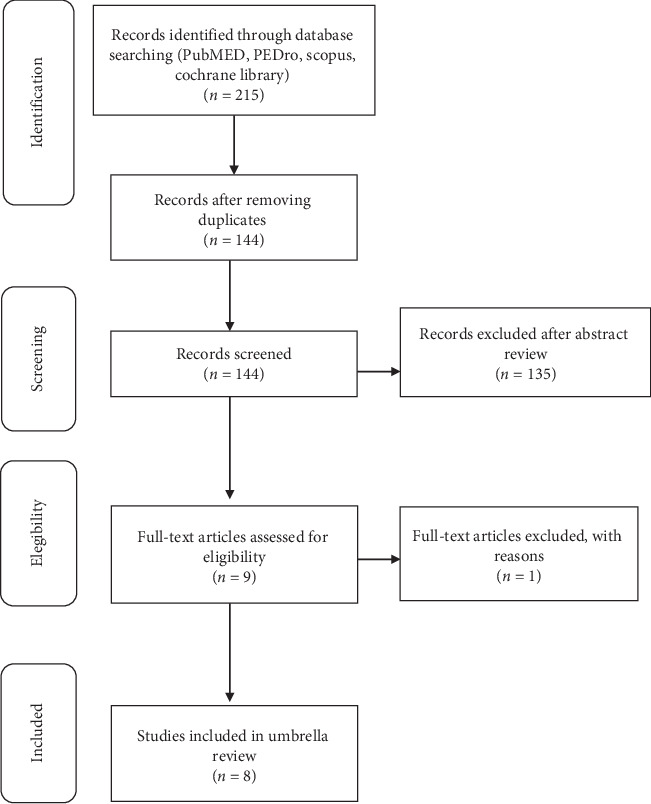
Flowchart of the included studies.

After duplicates were removed, the search resulted in 144 records; 135 records were screened on the basis of their titles and abstracts, and one article was rejected because it was not a systematic review [33]. Eight papers (six systematic reviews and meta-analyses and two systematic reviews) were considered to be relevant for qualitative analysis [21, 22, 27–32] because they fulfilled the inclusion criteria (Figure 1). The key features of the papers are summarized in Table 3; Table 4 summarizes the rehabilitation programs in each paper.

The aforementioned studies examined how progressive training benefits muscle strength. Various assessment measures were used in these reviews, such as one RM (*one repetition maximum*; the maximum weight that can be lifted once [34]) and the dynamometer, which is considered the standard for testing muscle strength [35].

The first systematic review to examine the effects of resistance training in PD was by Brienesse and Bhalsing. The authors reviewed five studies, where they found that resistance training improves muscle strength and endurance, mobility, and performance on functional tasks and increases fat-free mass. This modality can be a part of the rehabilitation program in patients with mild-to-moderate PD [21]. In the same year, Lima et al. performed a systematic review and meta-analysis showing positive and moderate effect sizes on strength in people with PD (SMD = 0.50, CI 0.05–0.95; *I*^2^ = 10%). According to the authors, PRT should be a part of the exercise program to increase strength in mild and moderate PD and more studies are needed to determine the beneficial effects of PRT on physical performance, such as walking capacity [22].

In their systematic review and meta-analysis, Saltychev et al. examined whether there was evidence on the effectiveness of PRT on the spine and lower limb muscles. The authors analyzed 12 RCTs that studied the effects of PRT on fast and comfortable walking speed, the Timed Up and Go Test, the 6-min walk test, and maximum oxygen consumption. They found statistical but clinically insignificant results in favor of PRT and concluded that RCTs with larger sample sizes and longer follow-up periods that compared with other types of physical training were needed to make clinical recommendations [27].

Tillman et al. evaluated the impact of PRT on gait, balance, and leg strength in PD. The study showed significant results of PRT with regard to lower limb muscle strength (*p*=0.0014, SMD 1.42; 95% CI 464–2.376) in mild/moderate PD. The proposed PRT regimen lasted 8 to 24 weeks. The authors did not find conclusive evidence for PRT enhancing balance or gait [28]. Roeder et al. conducted a systematic review and meta-analysis in 2015, examining nine RCTs that evaluated muscle strength, focusing on knee extension and flexion by leg press. The most effective physical activity program was composed of PRT and aerobic/balance/stretching exercises [30].

A systematic review and meta-analysis by Uhrbrand et al. in 2015 evaluated resistance training, endurance training, and other training modalities in PD. The authors found strong evidence of improved resistance training strength (*Q* = 1.844, d.f. = 5, *p*=0.870). Moreover, resistance training prevented strength from deteriorating. The group did not observe a linear correlation between lower extremity strength and walking performance, but when muscle strength was low, walking performance decreased. The combination of resistance and endurance training can improve muscle strength and cardiorespiratory fitness [32].

Chung et al., in their systematic review and meta-analysis (401 participants in seven RCTs) conducted in 2016, confirmed the effectiveness of PRT in PD. According to the meta-analysis, PRT had positive effects on muscle strength (*p* < 0.001; 95% CI, 0.35–0.87), balance (*p*=0.01; 95% CI, 0.08 to 0.64), and motor disorders (*p* < 0.001; 95% CI, 0.21 to 0.75) but did not elicit gains in walking performance. The authors suggested that moderate-intensity resistance training be performed in group and in the patient's home [29].

A systematic review and meta-analysis by Cruickshank et al. examined papers on PD (six RCTs and three non-RCTs) and multiple sclerosis (five RCTs and two non-RCTs) and showed that strength training significantly improved muscle strength (15% to 83.2%) and mobility (11.4%) in PD patients. Furthermore, Cruickshank et al., in accordance with the American College of Sports Guidelines, recommended *“progressive submaximal strength training (whole-body single and multi-joint resistance exercises) on at least 2 nonconsecutive days per week for an hour under direct supervision (physiotherapist, exercise physiologist, strength and conditioning specialist).”* Moreover, strength training slowed disease progression in patients with mild-to-advanced disability; these data also suggested that strength exercise has positive impacts on the progression of PD at all disease stages. However, the authors expressed doubts about the beneficial effects of strength training in the advanced stages of PD, concluding that future trials should include patients with severe levels of disability [31].

## 4. Discussion

The aim of this umbrella review was to provide evidence that PRT improves strength in PD, and the results favor the inclusion of strengthening exercises in the rehabilitation of PD patients through the improvement in muscle strength, and patients performed better in ADLs. Considering that muscle weakness can be a primary symptom of PD [36], which contributes to postural instability and gait difficulties [37, 38] and has been identified as a secondary cause of bradykinesia [39], this is an important insight and emphasizes the value of PRT in the treatment of PD. Furthermore, since muscle weakness and bradykinesia in PD have the same neuro-physio-pathological mechanisms [40], PRT can increase the power-generating capacity of the muscle, thus directly affecting muscle weakness. Improvements in muscle strength and power also have a significant impact on bradykinesia [19] and could facilitate independence in the community, improve functional mobility, and reduce the risk of falls [33].

To maximize the benefits of PRT, one must follow three key points: (1) progressive overload, which is the gradual increase in physical stress to which the body is subjected during training, (2) specificity, which refers to how the body responds and adapts to different variables of the training program with reference to the objective that has been set, and (3) variation, which is a change in one or more variables in the program for the training stimulus to remain optimal [41, 42]. The latter concept is based on Selye's theory of “general adaptation syndrome,” which describes how the body adapts through three phases in response to the stress of training (shock, adaptation, and, without variation in the training stimulus, staleness) [43].

Exercise improves the health of the brain, including increased expression of neurotrophic factors, greater blood flow, altered immune response, increased neurogenesis, and altered metabolism [44]. Such changes may enhance the neuronal circuitry between the basal ganglia and its cortical and thalamic connections, ultimately improving motor, nonmotor, and cognitive behavior in patients with PD [45].

In which phase of PD could PRT be suggested? In six of eight studies [21, 22, 27–29, 31], the reported severity of the disease was mild to moderate; in the remaining two studies [30, 32], disease severity was not specified. These results suggest that progressive resistance exercises can be effective in PD patients who lack comorbidities and severe disability. Additionally, physical activity has been associated with increased survival rates of individuals with PD [46]. In their review, LaStayo et al. emphasized how the two properties that define eccentric muscle contractions, that is, the potential for high muscle strength production at a uniquely low energy cost, should be revisited as exercise countermeasures to muscle atrophy, weakness, and physical functional deficits in chronic diseases such as cardiac and obstructive pulmonary disease, cancer, and neurological conditions [47].

Which exercise protocol should be adopted with respect to PRT? The trials [21, 22, 27–32] in our study suggest that, on average, 10 weeks of progressive resistance exercises for the upper and lower limbs should be performed two to three times a week. The most frequent upper limb exercises are bicep curl, chest press, triceps extension, and dumbbell side raise. Lower limb exercises are leg press, leg curl, half squat, hip abductors, hip flexors, and lateral step-up [27, 28]. During physical therapy, the patient plays a central role, choosing with the therapist which exercises and how many series and repetitions they will perform together. From our analysis, two to three series for each exercise, composed of 8–12 repetitions, is ideal for a patient with mild-to-moderate PD [22, 27, 28, 31, 32]. The training session should last from 45 to 90 minutes, including the recovery time. All exercises are performed at 30% of 70% of one RM. The load is increased by 5% to 10% of one RM when the patient is able to perform 10 repetitions at 60% of one RM [29] (Table 5).

In support of the proposed PRT program, Shu et al. analyzed aerobic exercise for PD conditions, including treadmill training, dancing, walking, and Tai Chi, showing that aerobic exercise has immediate beneficial effects on improving motor action, balance, and gait in patients [48]. Also, Bhalsing et al. highlighted the importance of physical activity prescribed like nonpharmacological therapy to manage the inherent decline that is associated with PD, It is a beneficial, cost-effective, and low-risk intervention that improves the overall health with regard to motor and nonmotor symptoms [12].

A major limitation of these studies is their lack of the duration of recovery times, which is an important training feature. None of the studies dealt with postworkout follow-up, which could help establish a proper training session. The postworkout follow-up can give important data about the long-term effectiveness of PRT. Moreover, there was no detailed evidence about the value of PRT in fall prevention, given that it is a rehabilitation goal in PD. Further, the data on improved walking performance after PRT are not clear, and practitioners should know how PRT influences gait and balance in various stages of disability between patients [22, 27]. Good walking capacity allows the patient to have a minimum level of autonomy. Our analysis suggests that PRT should be a part of the rehabilitation program, in combination with other types of exercise to preserve cardiorespiratory fitness. In summary, these findings demonstrate the effectiveness of progressive training in mild-to-moderate PD. It would be appropriate to find a standard assessment for measuring strength that can lead to homogeneous results. Following a clear rehabilitation program could facilitate comparisons between studies. Further research is needed to clarify this issue and determined whether it could be helpful in the late stages of PD.

## 5. Conclusion

PD is a complex disease that can compromise physical performance. Positive evidence for physiotherapy is growing, showing beneficial impact on functional activities that involve gait, transfers, and balance [49]. For the PD patient, exercise has reported benefits for controlling motor and nonmotor symptoms, with the use of pharmacological interventions [50]. This umbrella review also shows that PRT has benefits particularly in the early stages of PD, with low-to-moderate impact training exercises. In general, exercises should be prescribed and encouraged in all PD patients. When an increase in physical activity is recommended, several specific factors should be considered for patients with PD: motor symptoms (bradykinesia, tremor, and dystonia), risk of falls, apathy, fatigue, depression, and cognitive dysfunction. Each of these symptoms can reduce participation and contribute to a more sedentary lifestyle in PD patients [8]. Doctors should encourage and motivate PD patients to exercise regularly from the time of diagnosis and provide guidance with respect to the positive effects of physical exercise for the body and brain.

## Figures and Tables

**Table 1:** Inclusion and exclusion criteria used for paper selection.

Inclusion criteria	Exclusion criteria
(i) Systematic review or meta-analysis	(i) Narrative review/original study
(ii). Published between 2009 and 2019	(ii) Published before 2009
(iii) English language	(iii) Other languages
(iv) Full text available	(iv) Full text not available
(v) Patients with Parkinson's disease (any level of severity)	(v) Populations with other diseases
(vi) Male and female	(vi) Aged <18 years old
(vii) Aged >18 years old	(vii) Rehabilitation program not including PRT/other intervention
(viii) Motor disorders rehabilitation management including PRT	

**Table 2:** Methodological quality.

	Q1	Q2	Q3	Q4	Q5	Q6	Q7	Q8	Q9	Q10	Q11
Saltychev et al. [27]	Y	Y	Y	Y	Y	Y	Y	Y	Y	Y	Y
Tillman et al. [28]	Y	Y	Y	Y	Y	Y	Y	Y	Y	Y	Y
Chung et al. [29]	Y	Y	Y	Y	Y	Y	Y	Y	Y	N	N/A
Roeder et al. [30]	Y	Y	Y	Y	Y	Y	Y	Y	Y	N/A	Y
Lima et al. [22]	Y	Y	Y	Y	Y	Y	Y	Y	Y	Y	Y
Cruisckshank et al. [31]	Y	Y	Y	Y	Y	N/A	Y	N/A	Y	Y	N
Uhrbrand et al. [32]	Y	Y	Y	Y	Y	N/A	N	Y	Y	Y	Y
Brienesse and Emerson [21]	Y	Y	N/A	N/A	Y	Y	Y	Y	Y	Y	Y

Legend: Q1 = Is the review question clearly and explicitly stated?; Q2 = Were the inclusion criteria appropriate for the review question?; Q3 = Was the search strategy appropriate?; Q4 = Were the sources and resources used to search for studies adequate?; Q5 = Were the criteria for appraising studies appropriate?; Q6 = Was critical appraisal conducted by two or more reviewers independently?; Q7 = Were there methods to minimize errors in data extraction?; Q8 = Were the methods used to combine studies appropriate?; Q9 = Was the likelihood of publication bias assessed?; Q10 = Were recommendations for policy and/or practice supported by the reported data?; Q11 = Were the specific directives for new research appropriate?; N = no, Y = yes; N/A = not applicable.

**Table 3:** Papers' key features.

Author and year	Type of study	Method to assess studies' quality	Finding	Limits
Saltychev et al. [27]	Systematic review and meta-analysis (12 RCTs)	Cochrane Collaboration's domain-based evaluation framework	No evidence for the superiority of PRT compared to other physical training	Some relevant studies could remain undetected because of the uncertain definition of PRT

Tillman et al. [28]	Systematic review and meta-analysis (7 RCTs)	PEDro scale	On measures of gait and balance no evidence to support or refute PRT prescription; in conjunction with balance and task-specific functional training to improve these measures	The reduced sample size, which has been selected for the feasibility of conducting a one-to-one, 6-week exercise intervention.

Chung et al. [29]	Systematic review and meta-analysis (7 RCTs)	PEDro scale	PRT (2-3 times per week, 8–10 weeks) can enhance strength, balance and motor symptoms in early—moderate PD.	No blinding of subjects, intervention therapist and outcome assessors in the most of the RCTs. Outcome evaluation over short-term (8–14 weeks)

Roeder et al. [30]	Systematic review and meta-analysis (9papers)	2 review authors using a customized form	Combining RT with other form of physical exercise could be most effective. Not enough data available to evidence-based guidelines for resistance training prescription	Published reports did not provide sufficient details for judgment, then bias from selective reporting of results and from allocation concealment was difficult to determine; Since much was unknown about the quality of most included studies, it impacts on conclusions drawn from this review which are not definitive.

Lima et al. [22]	Systematic review and meta—analysis (2 RCTs and 2 QRCTs.).	PEDro scale	Evidence to support PRT prescription in mild/moderate PD's patients and to implemented it as an ordinary therapy in PD.	Only 4 studies included

Cruisckshank et al. [31]	Systematic review and meta-analysis (6RCTs + 3 non-RCTs about PD and 5RCTs + 2 non-RCTs about MS)	PEDro scale	Benefit in strength after RT, it has a positive effect on clinical disease progression and mobility. High-quality trials are needed.	The heterogeneity of interventions and study outcomes in PD and MS trials

Uhrbrand et al. [32]	Systematic review and meta-analysis (9RCTs)	PEDro scale	Strong evidence in favor of muscle strength improving in PD. Inconsistent findings are about balance, walking performance and quality of life improving. PRT may positively impact UPDRS-III and quality of life.	Small sample size; short-term interventions (≤12 settimane)

Brienesse and Emerson [21]	Systematic review (3 RCTs and 2 non-RCTs.)	Modified version of PEDro scale	RCTs with a standardized and thorough reporting of intervention and functional outcomes are needed.	Methodological limitation in available papers (RCTs do not satisfy all of quality criteria); not well RT description/inadequate description of the training protocol.

RCTs (randomized control trials), UPDRS-III = Unified Parkinson's Disease Rating Scale-III, MS = Multiple Sclerosis., PD = Parkinson disease, PRT = progressive resistance training, RT = resistance training.

**Table 4:** Rehabilitation programs of the studies included in the review.

Rehab program	Saltychev [27]	Tillman [28]	Chung [29]	Roeder [30]	Lima [22]	Cruisckshank [31]	Uhrbrand [32]	Brienesse and Emerson [21]
Case group	Leg press, leg curl, calf press, trunk's exercises, half squat, hip abductors, hip flexors, lateral	Leg press, leg curl, calf press, trunk's exercises, half squat, hip abductors, hip flexors, lateral	Upper limb (30–40% 1RM) Lowe limb (50–60% 1RM)	Leg press, leg curl, trunk's exercises	Leg press, leg curl, half squat, bicep curl	Leg press, leg curl, half squat, hip abductors, hip flexors, lateral step-up, bicep curl, trunk's exercise	Leg press, leg curl, half squat, hip abductors, hip flexors, lateral step-up, bicep curl.	No specified exercises
	N° sessions/week: 2-3 (60–90 minutes/each)	N° sessions/week: 2–3 (45–90 minutes/each)	N° sessions/week: 2-3 (45–90 minutes/each)	N° sessions/week: 2-3 (45–60 minutes/each).	N° sessions/week: 2-3 (45–60 minutes)	N° sessions/week: 2-3 (45–60 minutes)	N° sessions/week: 2-3 (45–60 minutes)	N° sessions/week: 2-3 (45–60 minutes)
	N° exercises/lesson: 8–12	N° exercises/lesson: 8–12	N° exercises/lesson: 10–12	N° exercises/lesson: 8–10	N° exercises/lesson: 8–12	N° exercises/lesson: 8–12	N° exercises/lesson: 8–12	N° exercises/lesson: 8–15
	Series: 2-3/type of exercise	Series: 2-3/type of exercise	Series: 2-3/type of exercise	Series: 2-3/type of exercise	Series: 2-3/type of exercise	Series: 2-3/type of exercise	Series: 2-3/type of exercise	Series: 2-3/type of exercise
	Recovery time: no specified	Recovery time: No specified	Recovery time: No specified	Recovery time: No specified	Recovery time: No specified	Recovery time: 2–5 minutes	Recovery time: No specified	Recovery time: No specified
	Period: 2/3 months	Period: 2/4 months	Period: 2/4 months	Period: 10/24 weeks	Period: 10/24 weeks	Period: 8/12 weeks	Period: 8/12 weeks	Duration: 8/12 weeks


Control group	Endurance and balance training + standard exercise	Standard exercise + treadmill training	Stretching and BT + treadmill training	Standard exercise	Standard exercise and balance training	Standard exercise and treadmill training	Shame therapy, endurance training and standard exercise	Stretching, Standard Exercise and movement strategy training

**Table 5:** Example of progressive resistance exercises.

Exercises	Upper limb (bicep curl, chest press, triceps extension, dumbbell side raise) Lowe limb (leg press, leg curl, half squat, hip abductors, hip flexors, lateral step-up)
N° week sessions	2-3
N° exercises for lesson	6–10
Series	2-3 series of 10 repetitions
Recovery time	No length mentioned
Rehabilitation program time	10–14 weeks

## References

[1] Kalia L. V., Lang A. E. (2015). Parkinson’s disease. *The Lancet*.

[2] Chapuis S., Ouchchane L., Metz O., Gerbaud L., Durif F. (2005). Impact of the motor complications of Parkinson’s disease on the quality of life. *Movement Disorders*.

[3] Braak H., Braak E. (2000). Pathoanatomy of Parkinson’s disease. *Journal of Neurology*.

[4] Braak H., Tredici K. D., Rüb U., de Vos R. A. I., Jansen Steur E. N. H., Braak E. (2003). Staging of brain pathology related to sporadic Parkinson’s disease. *Neurobiology of Aging*.

[5] Tysnes O.-B., Storstein A. (2017). Epidemiology of Parkinson’s disease. *Journal of Neural Transmission*.

[6] Lamont R. M., Morris M. E., Woollacott M. J., Brauer S. G. (2012). Community walking in people with Parkinson’s disease. *Parkinson’s Disease*.

[7] Wijlhuizen G. J., de Jong R., Hopman-Rock M. (2007). Older persons afraid of falling reduce physical activity to prevent outdoor falls. *Preventive Medicine*.

[8] Bloem B. R., van Vugt J. P., Beckley D. J. (2001). Postural instability and falls in Parkinson’s disease. *Advances in Neurology*.

[9] Goodwin V. A., Richards S. H., Henley W., Ewings P., Taylor A. H., Campbell J. L. (2011). An exercise intervention to prevent falls in people with Parkinson’s disease: a pragmatic randomised controlled trial. *Journal of Neurology, Neurosurgery & Psychiatry*.

[10] Abbruzzese G., Marchese R., Avanzino L., Pelosin E. (2016). Rehabilitation for Parkinson’s disease: current outlook and future challenges. *Parkinsonism & Related Disorders*.

[11] Mirelman A., Maidan I., Deutsch J. E. (2013). Virtual reality and motor imagery: promising tools for assessment and therapy in Parkinson’s disease. *Movement Disorders*.

[12] Bhalsing K. S., Abbas M. M., Tan L. C. S. (2018). Role of physical activity in Parkinson’s disease. *Annals of Indian Academy of Neurology*.

[13] Tajiri N., Yasuhara T., Shingo T. (2010). Exercise exerts neuroprotective effects on Parkinson’s disease model of rats. *Brain Research*.

[14] Fayyaz M., Jaffery S. S., Anwer F., Zil-E-Ali A., Anjum I. (2018). The effect of physical activity in Parkinson’s disease: a mini-review. *Cureus*.

[15] Keus S. H. J., Bloem B. R., Verbaan D. (2004). Physiotherapy in Parkinson’s disease: utilisation and patient satisfaction. *Journal of Neurology*.

[16] Yarnall A., Rochester L., Burn D. J. (2011). The interplay of cholinergic function, attention, and falls in Parkinson’s disease. *Movement Disorders*.

[17] Keus S. H. J., Bloem B. R., Hendriks E. J. M., Bredero-Cohen A. B., Munneke M. (2007). Evidence-based analysis of physical therapy in Parkinson’s disease with recommendations for practice and research. *Movement Disorders*.

[18] Morris M. E., Iansek R., Kirkwood B. (2009). A randomized controlled trial of movement strategies compared with exercise for people with Parkinson’s disease. *Movement Disorders*.

[19] Dibble L. E., Hale T. F., Marcus R. L., Droge J., Gerber J. P., LaStayo P. C. (2006). High-intensity resistance training amplifies muscle hypertrophy and functional gains in persons with Parkinson’s disease. *Movement Disorders*.

[20] Taylor N. F., Dodd K. J., Damiano D. L. (2005). Progressive resistance exercise in physical therapy: a summary of systematic reviews. *Physical Therapy*.

[21] Brienesse L. A., Emerson M. N. (2013). Effects of resistance training for people with Parkinson’s disease: a systematic review. *Journal of the American Medical Directors Association*.

[22] Lima L. O., Scianni A., Rodrigues-de-Paula F. (2013). Progressive resistance exercise improves strength and physical performance in people with mild to moderate Parkinson’s disease: a systematic review. *Journal of Physiotherapy*.

[23] Kanegusuku H., Silva-Batista C., Peçanha T. (2017). Effects of progressive resistance training on cardiovascular autonomic regulation in patients with Parkinson disease: a randomized controlled trial. *Archives of Physical Medicine and Rehabilitation*.

[24] Gabriel D. A., Kamen G., Frost G. (2006). Neural adaptations to resistive exercise. *Sports Medicine*.

[25] Carroll T. J., Benjamin B., Stephan R., Carson R. G. (2001). Resistance training enhances the stability of sensorimotor coordination. *Proceedings of the Royal Society of London. Series B: Biological Sciences*.

[26] Joanna Briggs Institute (2017). *Checklist for Systematic Reviews and Research syntheses, Critical Appraisal Tools*.

[27] Saltychev M., Bärlund E., Paltamaa J., Katajapuu N., Laimi K. (2016). Progressive resistance training in Parkinson’s disease: a systematic review and meta-analysis. *BMJ Open*.

[28] Tillman A., Muthalib M., Hendy A. M. (2015). Lower limb progressive resistance training improves leg strength but not gait speed or balance in Parkinson’s disease: a systematic review and meta-analysis. *Frontiers in Aging Neuroscience*.

[29] Chung C. L. H., Thilarajah S., Tan D. (2016). Effectiveness of resistance training on muscle strength and physical function in people with Parkinson’s disease: a systematic review and meta-analysis. *Clinical Rehabilitation*.

[30] Roeder L., Costello J. T., Smith S. S., Stewart I. B., Kerr G. K. (2015). Effects of resistance training on measures of muscular strength in people with Parkinson’s disease: a systematic review and meta-analysis. *PLoS One*.

[31] Cruickshank T. M., Reyes A. R., Ziman M. R. (2015). A systematic review and meta-analysis of strength training in individuals with multiple sclerosis or Parkinson disease. *Medicine*.

[32] Uhrbrand A., Stenager E., Pedersen M. S., Dalgas U. (2015). Parkinson’s disease and intensive exercise therapy--a systematic review and meta-analysis of randomized controlled trials. *Journal of the Neurological Sciences*.

[33] David F. J., Rafferty M. R., Robichaud J. A. (2012). Progressive resistance exercise and Parkinson’s disease: a review of potential mechanisms. *Parkinson’s Disease*.

[34] Fleck S. J., Kraemer W. (2014). *Designing Resistance Training Programs*.

[35] Martin H. J., Yule V., Syddall H. E., Dennison E. M., Cooper C., Aihie Sayer A. (2006). Is hand-held dynamometry useful for the measurement of quadriceps strength in older people? A comparison with the gold standard biodex dynamometry. *Gerontology*.

[36] Kakinuma S., Nogaki H., Pramanik B., Morimatsu M. (1998). Muscle weakness in Parkinson’s disease: isokinetic study of the lower limbs. *European Neurology*.

[37] Inkster L. M., Eng J. J., MacIntyre D. L., Stoessl A. J. (2003). Leg muscle strength is reduced in Parkinson’s disease and relates to the ability to rise from a chair. *Movement Disorders*.

[38] Nallegowda M., Singh U., Handa G. (2004). Role of sensory input and muscle strength in maintenance of balance, gait, and posture in Parkinson’s disease. *American Journal of Physical Medicine & Rehabilitation*.

[39] Berardelli A., Rothwell J. C., Thompson P. D., Hallett M. (2001). Pathophysiology of bradykinesia in Parkinson’s disease. *Brain*.

[40] Lang A. E., Lozano A. M. (1998). Parkinson’s disease. *New England Journal of Medicine*.

[41] Kraemer W. J., Ratamess N. A., Flanagan S. D., Shurley J. P., Todd J. S., Todd T. C. (2017). Understanding the science of resistance training: an evolutionary perspective. *Sports Medicine*.

[42] Kraemer W. J., Ratamess N. A. (2004). Fundamentals of resistance training: progression and exercise prescription. *Medicine & Science in Sports & Exercise*.

[43] Selye H. (1976). Forty years of stress research: principal remaining problems and misconceptions. *Canadian Medical Association Journal*.

[44] Hirsch M. A., Iyer S. S., Sanjak M. (2016). Exercise-induced neuroplasticity in human Parkinson’s disease: what is the evidence telling us?. *Parkinsonism & Related Disorders*.

[45] Petzinger G. M., Fisher B. E., McEwen S., Beeler J. A., Walsh J. P., Jakowec M. W. (2013). Exercise-enhanced neuroplasticity targeting motor and cognitive circuitry in Parkinson’s disease. *The Lancet Neurology*.

[46] Kuroda K., Tatara K., Takatorige T., Shinsho F. (1992). Effect of physical exercise on mortality in patients with Parkinson’s disease. *Acta Neurologica Scandinavica*.

[47] LaStayo P., Marcus R., Dibble L., Frajacomo F., Lindstedt S. (2013). Eccentric exercise in rehabilitation: safety, feasibility, and application. *Journal of Applied Physiology*.

[48] Shu H. F., Yang T., Yu S. X. (2014). Aerobic exercise for Parkinson’s disease: a systematic review and meta-analysis of randomized controlled trials. *PLoS One*.

[49] Domingos J., Keus S. H. J., Dean J. (2018). The European physiotherapy guideline for Parkinson’s disease: implications for neurologists. *Journal of Parkinson’s Disease*.

[50] Ramaswamy B., Jones J., Carroll C. (2018). Exercise for people with Parkinson’s: a practical approach. *Practical Neurology*.

